# Persistent Postictal Central Apnea in Focal Seizures: Incidence, Features, and Imaging Findings

**DOI:** 10.1212/WNL.0000000000213856

**Published:** 2025-07-22

**Authors:** Stefano Meletti, Margherita Burani, Alice Ballerini, Giada Giovannini, Elisa Micalizzi, Niccolò Orlandi, Lisa Taruffi, Niccolò Biagioli, Simona Scolastico, Laura Madrassi, Matteo Pugnaghi, Anna Elisabetta Vaudano

**Affiliations:** 1 Department of Biomedical, Metabolic and Neural Sciences, University of Modena and Reggio Emilia, Italy;; 2 Neurophysiology Unit and Epilepsy Centre, Neuroscience Department, University of Modena, Italy;; 3 Neurophysiology Unit, Department of Neuroscience, IRCCS San Martino Hospital, Genoa, Italy; and; 4 Clinical and Experimental Medicine PhD Program, Department of Biomedical, Metabolic and Neural Sciences, University of Modena and Reggio Emilia, Modena, Italy.

## Abstract

**Background and Objectives:**

Postconvulsive central apnea has emerged as a contributor to sudden unexplained death in epilepsy. The aim of this study was to evaluate the incidence and characteristics of postictal central apnea (PICA) in focal seizures. The secondary aim was to analyze morphometric features of the amygdala and other subcortical structures involved in autonomic control.

**Methods:**

We prospectively enrolled consecutive patients admitted to the Epilepsy Monitoring Unit at Modena Academic Hospital (Italy) from April 2020 to December 2023. Inclusion criteria were as follows: (1) age older than 13 years; (2) at least 1 focal-onset seizure recorded during long-term video-EEG monitoring (LTVEM) with cardiorespiratory polygraphy. For each seizure, the presence of ictal central apnea (ICA) and/or PICA and its features were evaluated. Amygdala, hippocampus, thalamus, brainstem, and cerebellum volumetry were compared in patients with ICA/PICA with respect to healthy controls and patients with focal seizures without peri-ictal breathing disorders.

**Results:**

A total of 69 patients (mean age 35.7 years; 42% female) with 406 focal-onset seizures were analyzed. ICA was recorded in 71 seizures (17%) in 27 patients. PICA was recorded in 24 seizures in 12 patients (10 with temporal lobe epilepsy) corresponding to 5.9% of all recorded seizures. Notably, PICA was observed only in seizures showing ictal apnea (in 33.8%). In 11 seizures with PICA, a single apneic event starting in the ictal and extending to the postictal period was observed. In 13 seizures, multiple apneic events were present in the postictal period (range 2–8). Seizures with PICA showed a longer peri-ictal apnea time (mean 75 seconds vs 40 seconds; *p* = 0.007) and a longer time to restore a regular rhythmic breathing after seizure termination (mean 173 seconds vs 42 seconds; *p* < 0.001) than seizures with self-limiting ictal apnea. Amygdala volumes ipsilateral to the epileptogenic zone were larger in patients with ICA/PICA compared with controls and patients without seizure-related apnea.

**Discussion:**

PICA occurs in approximately 6% of focal seizures and is associated with extended apnea time and an enlarged amygdala ipsilaterally to the epileptogenic zone. Our data support the existence of a continuum from ictal to PICA and highlight the importance of cardiorespiratory recordings in LTVEM.

## Introduction

Sudden unexpected death in epilepsy (SUDEP) has a global incidence ranging from 0.22 to 1.2 per 1,000 individuals per year,^1^ accounting for up to 10%–50% of all deaths in individuals with medically refractory epilepsy.^2^ Growing evidence suggests that most of the SUDEP cases occur after tonic-clonic seizures due to respiratory failure.^3,4^ Critical insights into SUDEP and near-SUDEP have been gleaned from rare cases observed in epilepsy monitoring units, where electroencephalographic, cardiac, and respiratory functions can be assessed simultaneously.^4-7^ These studies collectively indicate that postconvulsive central apnea is a major contributor to SUDEP.^8^ However, the underlying mechanisms leading to postictal apnea remain unknown.

In contrast to postconvulsive apnea, ictal central apnea (ICA) occurs during a seizure and is more common but generally less severe. ICA is estimated to occur in 33%–40.5% of all seizures.^9-12^ Studies involving adult and pediatric patients with epilepsy undergoing intracranial EEG (iEEG) and continuous respiratory monitoring have shown that seizure propagation to the amygdala, as well as amygdala electrical stimulation, is associated with ICA.^13-17^ These findings suggest that the amygdala is functionally connected to the brainstem respiratory network, can inhibit breathing, and plays a key role in ictal apnea. However, they also raise important questions about the potential relationship between the amygdala and postictal apnea associated with SUDEP, in particular, of whether ictal and postictal apnea are related or distinct phenomena.^18,19^ It also prompts consideration of whether postictal apnea occurs exclusively after convulsive seizures or if it can also result from focal limiting seizures.

Recently, a multimodal approach to study brain mechanisms of breathing control in patients undergoing iEEG^20^ indicated that amygdala seizures induced by electrical stimulation can cause prolonged postictal breathing loss, persisting well beyond the end of seizures or stimulation without any subjective sensation of air hunger. We recently reported persistent ictal and postictal breathing alterations in focal seizures due to *DEPDC5* pathogenic variants,^21^ a genetic epilepsy associated with an increased SUDEP risk. These findings contrast with previous results from spontaneous seizures recorded during noninvasive long-term video-EEG monitoring (LTVEM), where ICA persisting into the postictal period was extremely rare (3% of seizures showing ICA).^11^

Evaluating the incidence and characteristics of postictal central apnea (PICA) in focal seizures is crucial for enhancing our understanding of the neural mechanisms that lead to seizure-related respiratory dysfunction. Therefore, we conducted a detailed analysis of breathing alterations during the postictal period of prospectively acquired focal seizures in LTVEM at our center. Finally, we evaluated the role of amygdala and several subcortical structures in patients with ictal/postictal apnea by means of MRI morphometry approaches.

## Methods

### Patients and Clinical Setting

We prospectively enrolled consecutive patients admitted to the Epilepsy Monitoring Unit (EMU) at the Baggiovara Civil Hospital, Modena Academic Hospital (Modena, Italy), from April 2020 to December 2023. Patients were admitted to the EMU for both diagnostic purpose and presurgical evaluation.

All the patients included in this study underwent the following: prolonged video-EEG monitoring, including at least an overnight polysomnography; a high-field (3T) brain MRI study with a dedicated epilepsy protocol; CSF analysis when clinically indicated (suspect of autoimmune encephalitis, infectious, inflammatory causes).

Inclusion criteria for this study were as follows: (1) age older than 13 years; (2) at least 1 focal-onset seizure recorded during LTVEM with cardiorespiratory polygraphy. Patients were excluded if no focal-onset seizure was recorded during EMU admission and/or they experienced only psychogenic nonepileptic seizures.

For each patient, we collected the following electroclinical and demographic data: age at the time of admission, sex, age at epilepsy onset, disease duration, the hemisphere of the seizure onset, family history of epilepsy, and febrile seizures in medical history. In addition, we considered the number of seizures recorded, the state of vigilance at the seizure onset, brain MRI findings, and etiology. According to the last International League Against Epilepsy (ILAE) classification proposal,^22^ the etiology was classified as structural, genetic, metabolic, autoimmune, and unknown. The drug-response status of the patient at the time of EMU admission was defined according to the ILAE definition of drug resistance.^23^

### Cardiorespiratory and Video-EEG Monitoring

Every patient underwent a LTVEM with a 10-20 EEG system (Nihon Kohden Neurofax EEG-1200, Model JE-120) integrated with a standard precordial single-channel electrocardiogram, pulse oximetry for peripheral oxygen saturation (SpO_2_) measurement, and thoracoabdominal belt for respiratory inductance plethysmography as previously detailed.^12,21^ Seizures with poor-quality polygraphic recordings and artifact precluding evaluation of respiratory movements and oxygen saturation were discarded. For this study, we analyzed focal-onset seizures without secondary bilateral tonic-clonic evolution.

ICA was considered as a respiratory arrest of 5 or more seconds visible on the polygraphic respiratory channel, preceded by stable breathing for at least 5 seconds, and confirmed by visual inspection of the recorded video.^12,24,25^

PICA was considered immediate, if no breath was taken for at least 5 seconds after the seizure end, or delayed, when apnea occurred after at least 1 breath detected after the seizure end.^8^

Beyond ICA and PICA as defined above, we also evaluated the presence of breath alterations during the ictal and postictal period consistent with the definition of “ataxic breathing” including the following: alteration in respiratory rhythm generation, paradoxical breathing, inspiratory efforts with 2–3 peaks, random brief apneas (1–4 seconds), and no regularity.

A more thorough data analysis regarding patients with seizure-related apnea was performed collecting apnea duration, hypoxemia (duration, nadir and degree of oxygen desaturation), apnea awareness, and heart rate changes. Hypoxemia was defined as a drop of SpO_2_ value below 95% and classified as mild 90%–94%, moderate 75%–89%, or severe <75%.^11,12^ For patients who manifested seizure-related apnea, mean oxygen saturation preceding the seizure onset by 5 minutes was calculated. Tachycardia and bradycardia were, respectively, defined as heart rate >100 beats per minute and <60 beats per minute, or a >20% deviation from baseline.^11,12^

### MRI Acquisition and Postprocessing

We adopted surface-based morphometry of subcortical structures (amygdala, hippocampus, thalamus, brainstem, cerebellum) to explore volume differences among patients with seizure-related breathing disorders vs (1) patients without seizure-related breathing disorders and (2) healthy controls. For this last comparison, an imaging data set of sex/age-matched healthy controls who underwent a structural MRI scan on the same scanner and with the identical protocol was used as reference. Details of the MRI acquisition and postprocessing pipeline are described in the eMethods and eFigure 1, according to previous studies by our group.^26-33^

### Statistical Analysis

#### Clinical Variables

Summary statistics were reported as mean ± SD (median, range). Statistical significance for intergroup differences was assessed with the Pearson χ^2^ test and Fisher exact tests for dichotomous or nominal variables. The Spearman correlation was used to determine correlation between continuous non-normally distributed variables. The level of significance was set at *p* < 0.05. The statistical analysis was performed with SPSS (version 27; IBM Corp., Armonk, NY).

#### Morphometric MRI Analyses

To explore volume differences of the analyzed subcortical structures across groups, a series of multivariate analyses of covariance and relative post hoc analyses have been conducted. We searched for subcortical volume differences across the following groups:

1. PICA/ICA vs healthy controls.
2. Patients with seizure-related breathing disorders (PICA/ICA) vs patients without seizure-related apnea (no-ICA group).
3. Patients with PICA vs patients showing ICA only.

Age, sex, and intracranial volume (ICV) were included as covariates in all comparisons. Statistical significance for all tests was set at *p* < 0.05 and adjusted for multiple comparisons. *p* Values were considered significant when surviving a 5% false discovery rate (FDR) correction.

### Standard Protocol Approvals, Registrations, and Patient Consents

The study was approved by the local Ethical Committee of Area Vasta Emilia Nord (NET-2013-02355313 No. 155/14; No. 238/23). Patients gave written informed consent for the use of their clinical records in this study. The study was conducted in accordance with the World Medical Association Declaration of Helsinki.

### Data Availability

Data will be shared on reasonable request to the corresponding author.

## Results

**Figure 1:**
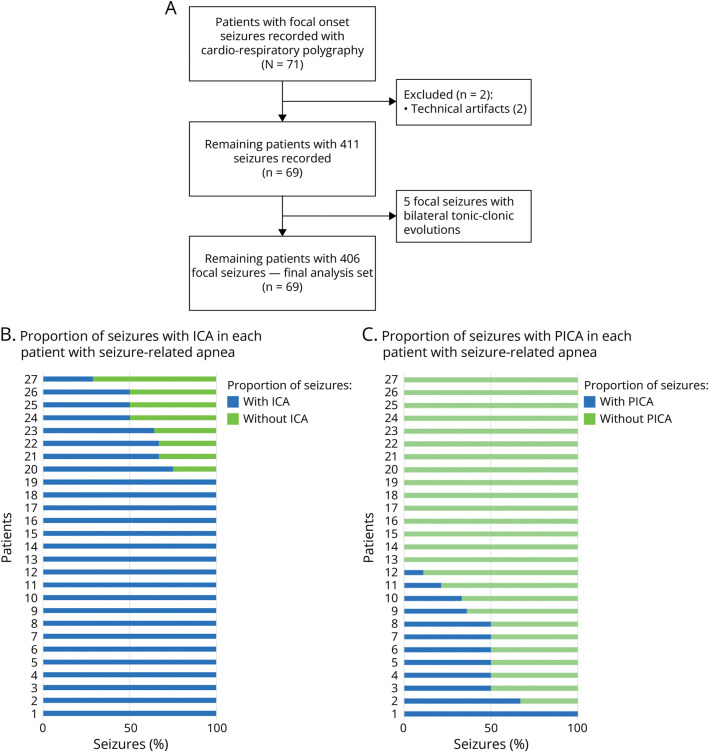
Study Flowchart (A) Flowchart of the study population according to inclusion/exclusion criteria (Methods in text). (B) Proportion of seizures with ICA of all captured seizures per individual patient in whom we recorded an ictal apnea event (n = 27). In most patients (from 1 to 19), ICA was observed in all seizures. (C) Proportion of seizures with PICA in each patient in whom we recorded at least 1 episode of ictal apnea (n = 27). Persistent postictal apnea was observed in 12 patients, showing intrapatient variability among seizures. ICA = ictal central apnea; PICA = postictal central apnea.

During the study period, 71 patients with focal-onset seizures fulfilled the inclusion criteria. However, owing to technical artifact, 2 patients were discarded from subsequent analyses. Therefore, 69 patients (42% female; mean age 35.7 years) with 411 seizures were enrolled. Five focal seizures with bilateral tonic-clonic evolution were recorded and then excluded from subsequent analyses; therefore, 406 focal seizures were finally included in the analysis set (study flowchart in Figure 1A). None of the patients had a history of snoring or sleep apnea, and overnight sleep polygraphy did not show obstructive apnea in any patient.

### ICA in Focal Seizures

First, we looked for the occurrence of ictal apnea, which was present in 17.5% of the total recorded seizures (71 of 406) and in 39% of the patients (27 of 69). Table 1 provides the electroclinical features of patients with and without ICA. No differences in age, age at epilepsy onset, epilepsy duration, drug response to antiseizure medications (ASMs), etiology, and side of seizure onset were observed between groups. Patients with ICA showed a higher frequency of temporal lobe seizure onset (85%) compared with patients without ICA (52%; *p* = 0.01). We conducted a secondary analysis on temporal lobe seizures in patients with (n = 23) and without (n = 22) ictal apnea. We did not find a significant difference in seizure duration between the 2 groups (median duration: 72 seconds in seizures without apnea vs 74 seconds in seizures with apnea).

**Table 1:** Characteristics of Patients With and Without ICA

	All	With ictal apnea	Without seizure-related apnea	Statistics	*p* Value
No. of patients	69	27	42		
No. of recorded seizures	406	71	335		
No. of recorded seizures per patient, mean (SE)	5.9 (2.8)	3.5 (1.9)	7.5 (3.0)	*t* = 2.05	0.043
Sex, n (%)				χ^2^ = 0.06	0.93
Female	29 (42)	12 (44)	17 (40)		
Male	40 (58)	15 (56)	25 (60)		
Age, y, mean (SE)	35.7 (1.7)	33.4 (2.4)	37.2 (2.4)	*t* = 1.06	0.28
Age at epilepsy onset, y, mean (SE)	22 (2.7)	20.1 (2.6)	23 (2.3)	*t* = 0.75	0.45
Epilepsy duration, mean (SE)	13.8 (1.7)	12.8 (2.9)	14.3 (2.1)	*t* = 0.42	0.66
Drug resistance, n (%)				χ^2^ = 1.24	0.26
Yes	47 (68)	21 (78)	26 (62)		
No	22 (32)	6 (22)	16 (38)		
No. of ASMs,^a^ mean (SE)	2.1 (1.1)	1.7 (0.1)	2.3 (0.1)	*t* = 3.40	0.0007
Etiology, n (%)				χ^2^ = 0.08	0.93
Structural	43 (62)	17 (63)	26 (62)		
Unknown	19 (28)	8 (30)	11 (26)		
Genetic	3 (4)	2 (7)	1 (2)		
Metabolic	2 (3)	0 (0)	2 (4)		
Autoimmune	2 (3)	0 (0)	2 (4)		
Seizure-onset zone, n (%)				χ^2^ = 6.41	0.01
Temporal lobe	45 (65)	23 (85)	22 (52)		
Extratemporal	24 (35)	4 (15)	20 (48)		
Hemisphere of seizure onset, n (%)				χ^2^ = 0.61	0.43
Right	36 (52)	12 (44)	24 (57)		
Left	33 (48)	15 (56)	18 (43)		

Abbreviations: ASM = antiseizure medication; ICA = ictal central apnea.

a The number of ASMs are referred to the day of each recorded seizure here. This was feasible by means of the electronic hospital system that tracks day by day the patient's treatment, allowing us to record the number of ASMs for each day in whom a seizure event was recorded.

As far as the “seizure burden” during the LTVEM, we calculated the average number of seizures per patient in patients with and without seizure-related disordered breathing, observing a higher number of seizures/patient in patients without ICA (*p* < 0.05) (Table 1). We also looked at patients with a high seizure burden during the LTVEM (>10 recorded seizures) that was present in 9 of 42 patients (21%) in the no-ICA group compared with 3 of 27 (11%) in the ICA group. Thus, we had no evidence for a higher “seizure burden” in patients with seizure-related disordered breathing. The mean number of ASMs was 1.7 for seizures with ICA (n = 71) and 2.3 for seizures without ICA (n = 335; *p* < 0.001).

Figure 1B shows the proportion of seizures with ICA of all captured seizures per individual with ictal apneic event.

### PICA in Focal Seizures

Postictal episodes of breathing loss were recorded in 12 patients (10 with temporal lobe epilepsy [TLE]) and in 24 seizures. The proportion of seizures with PICA in patients in whom we recorded at least 1 episode of ictal apnea was more variable (Figure 1C). eTable 1 presents the details of patients showing PICA while Table 2 provides the features of breathing alterations in patients with self-limiting ICA and in patients with PICA.

**Table 2:** Breathing Alterations in Seizures With ICA Only and in Seizures With Persistent PICA

	Total patients with seizure-related disordered breathing	Seizures with PICA	Seizures with ICA only	Stat	*p* Value
No. of patients	27	12	15		
No. of seizures	71	24	47		
Level of vigilance at seizure onset (69 seizures), n (%)				χ^2^ = 0.36	0.55
Wake	34 (49%)	12 (55%)	22 (47%)		
Sleep/stupor	35 (51%)	10 (45%)	25 (53%)		
Seizure duration (EEG), s, mean ± SE [median, range]	108.1 ± 13.3 [74.5; 28–780]	90.7 ± 18.1 [65.5; 29–426]	116.6 ± 18.2 [76; 28–780]	MW	0.242
Peri-ictal apnea duration, s, mean ± SE [median, range]	51.6 ± 6.1 [39; 5–331]	74.6 ± 13.9 [55; 15–331]	39.8 ± 5.1 [30; 5–180]	MW	0.007
Time to recovery of regular rhythmic breathing after seizure termination, s, mean ± SE [median, range]	85.9 ± 13.7 [36; 0–600]	172.7 ± 28.7 [130.5; 17–600]	41.6 ± 9.6 [13; 0–241]	MW	<0.001
Total time of peri-ictal respiratory alteration, s, mean ± SE [median, range]	197.2 ± 19.8 [124; 5–705]	292.6 ± 37.2 [282.5; 66–705]	148.5 ± 19.91 [107; 5–578]	MW	0.001
Duration of oxygen desaturation (<95%), s, mean ± SE [median, range]	61 ± 7.5 [50; 0–245]	69.6 ± 14.8 [54; 0–240]	54.7 ± 8.5 [49.5; 0–245]	MW	0.93
Nadir of oxygen desaturation, %, mean (median); range	87% (88%); 68%–99%	85% (86%); 68%–99%	88% (89%); 70%–99%	MW	0.17
Oxygen desaturation (60 seizures), n				χ^2^ = 1.03	0.8
No desaturation (≥95%)	10	2	8		
Mild (between 90% and 94%)	9	3	6		
Moderate (between 75% and 89%)	36	13	23		
Severe (<75%)	5	2	3		

Abbreviations: ICA = ictal central apnea; PICA = postictal central apnea.

**Figure 2:**
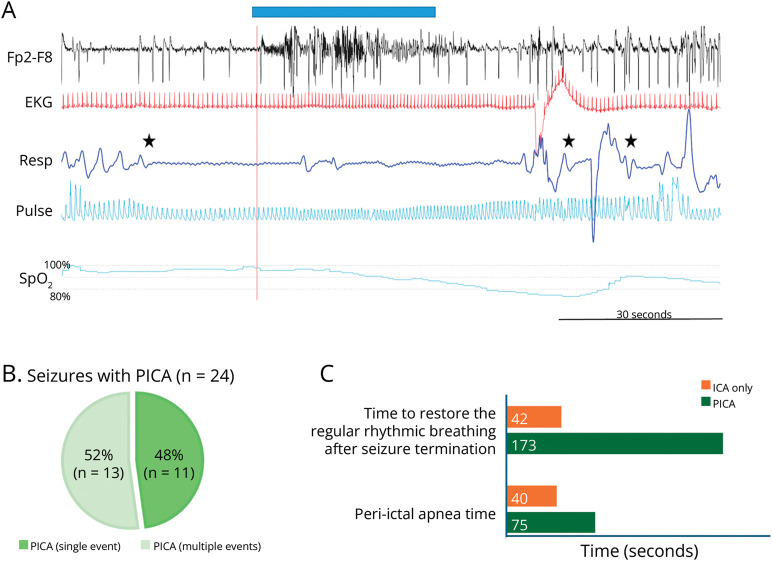
Features of Ictal and Postictal Apnea (A) A compressed 2-minute polygraphic recording of ictal apnea extending into the postictal period. The patient (MO#12) had a left frontotemporal lobe seizure (indexed here by the Fp1-F7 EEG channel). The blue bar at the top marks scalp EEG seizure onset and offset. The black star marks the onset of apnea that precedes the EEG seizure onset by 20 seconds. Note that apnea persisted after the EEG seizure termination for approximately 15 seconds. Then, an irregular breathing pattern was evident with 2 more brief apnea episodes in the postictal period (black stars). Oxygen saturation shows a progressive decrease with a nadir below 80% during the postictal period. (B) Percentage of seizures with self-limiting ICA and wit persistent PICA among all seizures with breathing alterations. (C) Duration of breathing alterations in seizures with ICA and PICA (details in text). ICA = ictal central apnea; PICA = postictal central apnea; Resp = thoracoabdominal respiration; SpO_2_ = peripheral oxygen saturation.

**Figure 3:**
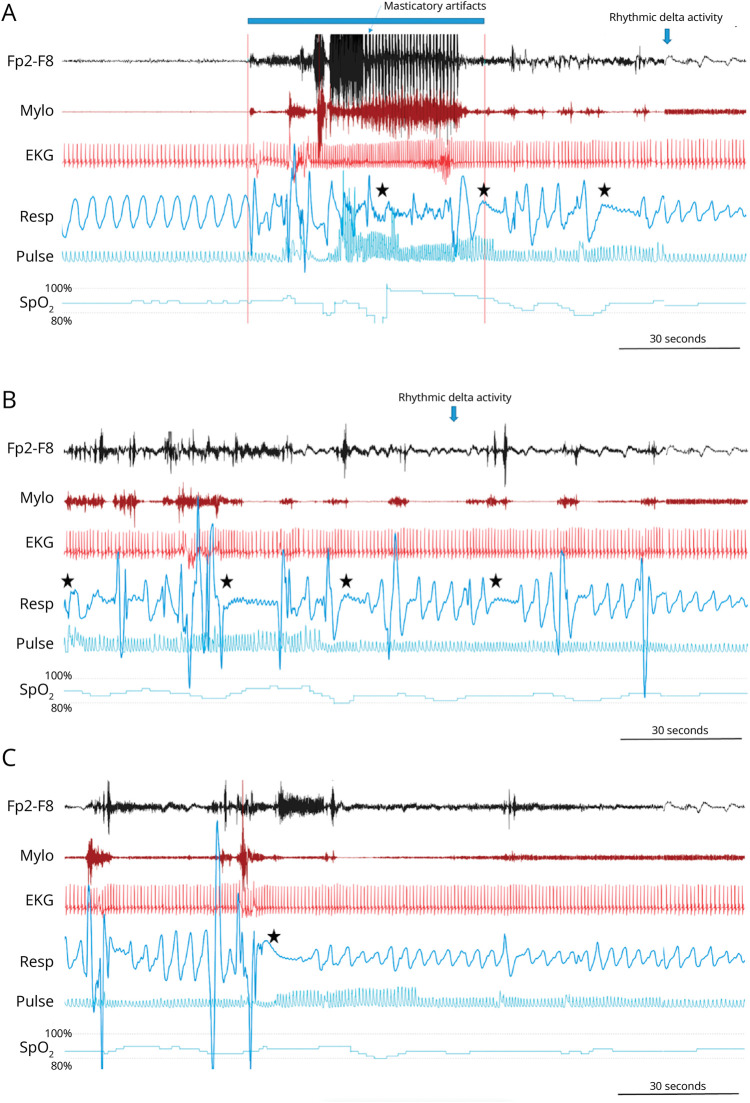
Persistent and Prolonged Postictal Apnea Episodes (A–C) Three consecutive compressed 3-minute peri-ictal polygraphic recording periods. The patient (MO#11) had a right frontotemporal lobe seizure (indexed here by the Fp2-F8 EEG channel). An ictal apnea followed by several postictal apnea episodes is evident (Resp channel) extending for several minutes after seizure termination. (A), EEG and the mylohyoid channels show muscle activity related to oroalimentary automatisms. The blue bar at the top marks scalp EEG seizure onset and offset. The black star marks the onset of ictal and postictal apnea episodes. Oxygen saturation showed a decrease between 85% and 80% that remained in this range until minute 8 of the postictal period. Milo = mylohyoid muscle; Resp = thoracoabdominal respiration; SpO_2_ = peripheral oxygen saturation.

Notably, PICA was never observed in seizures/patients without concomitant ICA. PICA was recorded in 33.8% of seizures showing ICA (24 of 71) and in 5.9% of all recorded seizures (24 of 406). In 11 of the recorded seizures showing PICA, we observed apneic event starting in the ictal and extending to the postictal period (Figure 2). In the remaining 13, multiple apneic events were observed in the postictal period (range 2–8) (Figure 3).

Seizures with PICA showed a longer peri-ictal apnea time (mean 75 seconds vs 40 seconds; *p* = 0.007) and a longer time to recovery of regular rhythmic breathing after seizure termination (mean 173 seconds vs 42 seconds; *p* < 0.001; Figure 2C) compared with seizures without PICA, despite similar median seizure duration (Table 2). The duration of oxygen desaturation and the oxygen nadir showed no significant differences in seizures with or without PICA, even if a trend for longer desaturation and lower nadir was recorded in seizures with PICA (Table 2). A significant correlation was present between apnea time and duration of oxygen desaturation (*p* < 0.001) in seizures with PICA. Severe oxygen desaturation (SpO_2_ <75%) was observed in 5 seizures. We never observed bradycardia episodes while tachycardia was observed in every seizure. Finally, we never observed a postictal generalized (or lateralized) EEG suppression in seizures with PICA.

### Awareness of Apnea in the Postictal Period and the Role of External Interventions

**Figure 4:**
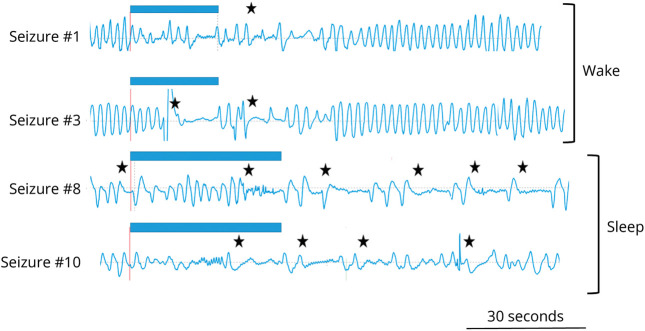
Seizure-Related Apnea During Wake and Sleep Respirogram during 4 different seizures in a young patient with focal left temporal lobe seizures due to herpes virus encephalitis (MO#01). Seizures 1 and 3 that occurred during wake were shorter (blue bars: scalp EEG seizure duration) and characterized by brief postictal apnea periods while seizures 8 and 10 that occurred during sleep showed prolonged and persistent apnea events. In all 4 seizures, the patient was not actively tested.

Previous studies^11,12,20,21^ underlined the unawareness of ictal apnea, even in those seizures with moderate/severe oxygen desaturation. Thus, we analyzed the video of the postictal period, when possible, to evaluate the patient's awareness of the seizure-related apnea. Considering seizures with ICA only, no patient reported apnea awareness when actively tested/interrogated (35 of 47 seizures). In 15 of 24 seizures with PICA, the patients were actively interrogated by the EEG technologist and in any seizures, the patient was aware of the apnea. In the remaining 8 seizures, the patients were not actively questioned about the apnea, but no patient showed sign of air hunger or subjective dyspnea. Notably, we observed that when the patient was awakened and/or had an interaction with an observer, breathing movements were clearly recorded by the thoracoabdominal belt. On the contrary, when the patient was left alone (as in some sleep-related seizures), the postictal breathing pattern was interrupted by several apneic events persisting for several seconds before the reappearance of a regular rhythmic breathing pattern (Figure 4 and eFigure 2). This implies that although the occurrence of PICA per se (Table 2) was not associated with sleep state or altered vigilance, the persistence and number and/or duration of apneic events in the postictal period was greater when the patient was alone, untested, and/or asleep. In our clinical setting, this was more common during night time because of the presence of reduced staff levels.

### Subcortical Structures and Amygdala Volumes

Twenty-two of 27 patients with ICA/PICA were included in the MRI analysis of subcortical structures. Two patients were excluded because of structural lesions involving the amygdala (i.e., ganglioglioma, herpes simplex encephalitis) and 3 were excluded because of MRI artifacts or low-quality imaging, ending with 22 patients enrolled: 12 with ICA (mean age 37.58 ± 13.59, 5 female) and 10 with PICA (mean age 30.33 ± 11.61, 5 female). A group of 31 patients with focal epilepsy without seizure-related breathing disorders (no-ICA, mean age 37.45 ± 14.51, 12 female) and a group of 30 healthy controls (mean age 32.20 ± 12.23, 15 female) were enrolled as comparison groups. There were no differences in age, sex, and ICV between the 4 groups (eTable 2). Hippocampal sclerosis on visual inspection of MRI scans was present in 1 patient with seizure-related apnea, whereas it was present in 5 patients of the no-ICA group.

**Figure 5:**
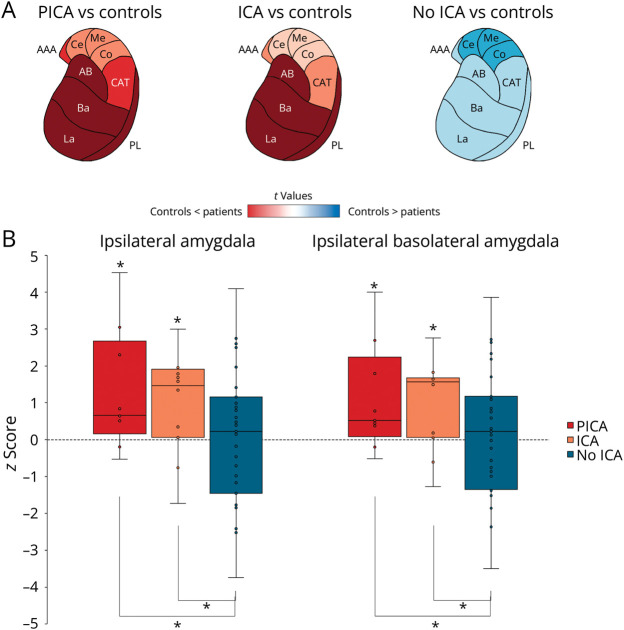
Amygdala Volumes in Patients With and Without Seizure-Related Apnea (A) The amygdala ipsilateral to the epileptic focus is presented. The comparisons between patients' groups and controls are reported in *t* values; blue colors represent an amygdala atrophy compared with controls while red colors indicate an increase in volume compared with controls. (B) The box-and-whisker plots show the z-scores of ipsilateral whole amygdala volume and ipsilateral basolateral amygdala volume in PICA, ICA, and no-ICA groups compared with controls. The boxes' central line marks the mean; upper and lower edges of the box (the hinges) mark the 25th and 75th percentiles (the central 50% of the values fall within the box). The open circles represent each individual patient's volume. The dashed line on the 0 indicates the mean volume of control population. Finally, the asterisks on the top of the boxes show the significant differences with the control group while lines and asterisks below the boxes show the significant differences across patient groups. AAA = anterior amygdaloid area; AB = accessory basal nucleus; Ba = basal nucleus; CAT = cortico-amygdaloid transition area; Ce = central nucleus; Co = cortical nucleus; ICA = ictal central apnea; La = lateral nucleus; Me = medial nucleus; PICA = postictal central apnea; PL = paralaminar nucleus.

No differences were observed in the brainstem and cerebellum across the groups. Similarly, no differences were found in the amygdala, hippocampus, and thalamic nuclei contralateral to the epileptogenic zone. However, we observed an increased volume of the whole amygdala ipsilateral to the epileptogenic zone (as well as in several amygdala subnuclei) in patients with ICA/PICA compared with both healthy controls (ICA: *F*(1,75) = 5.478, *p*_FDR_ = 0.037, PICA: *F*(1,75) = 5.478, *p*_FDR_ = 0.033) and the no-ICA group (ICA: *F*(1,75) = 5.478, *p*_FDR_ = 0.022, PICA: *F*(1,75) = 5.478, *p*_FDR_ = 0.012) (Figure 5 and eTable 3). No significant differences were found when comparing patients with self-limiting ICA with those with persistent PICA (eTable 3).

As far as the ipsilateral hippocampus, patients with ICA/PICA showed no differences compared with healthy controls. Notably, the no-ICA group showed a thinner hippocampus ipsilateral to the epileptogenic zone (particularly head and body) compared with healthy controls (eTable 4).

Finally, the thalamus ipsilateral to the epileptogenic zone showed no differences in ICA/PICA compared with healthy controls (eTable 5).

## Discussion

The main finding of this work is the documentation that postictal apnea is present in approximately 6% of focal seizures (without tonic-clonic evolution). In our series, postictal apnea is a phenomenon closely related to the presence of central ictal apnea as we have never observed postictal respiratory changes without a concomitant (and previous) central ictal apnea. This finding demonstrates how the 2 phenomena are intimately related and how the apneic phenomena persist for minutes after the end of the seizure (this happened in approximately 30% of seizures with ictal apnea).

We confirmed a prevalence of peri-ictal respiratory changes in patients with focal seizures arising from the temporal lobe, and particularly 83% of patients with postictal apnea had a TLE.^13,14,16,25,34^ An interesting result was that the number of focal seizures recorded during the LTVEM (seizure burden) was not associated with the occurrence of peri-ictal respiratory changes. By contrast, we observed that a lower number of ASMs were associated with the occurrence of seizures with ICA/PICA. This finding might suggest that a higher number of ASMs have a “protective” role regarding the occurrence of ICA/PICA.

From the quali-quantitative analysis of seizures with peri-ictal respiratory alterations, some data of particular interest emerged. First, we obtain the characteristics of PICA, which can present both as an “extension” of ictal apnea in the postictal period and also as 1 or more periods of respiratory arrest after the patient had resumed a respiratory rhythm for a few seconds. Noteworthy, in some seizures, the postictal respiratory irregularities continued for many minutes after the end of the seizure, thus testifying for a “fragility” of the brainstem respiratory centers persisting several minutes after the seizure.

A further element of interest, although difficult to quantify, is how respiratory arrest in the postictal period can be resolved simply because of a change in the patient's level of vigilance. When the patient is questioned, or awakened by an examiner, it seems clear that the apnea stops. This means that patients were able to override apnea, confirming that apnea was not caused by impairment in respiratory musculature or airway obstruction (e.g., due to laryngospasm). This phenomenon, which has already been described for apneic events induced by cerebral electrical stimulation of the amygdala,^20^ is relevant because the respiratory alterations may be more pronounced (and perhaps more dangerous) in absence of external stimulation. From this point of view, our data document how the state of vigilance per se at the seizure onset is not decisive for the onset of ICA/PICA while the patient's state of vigilance in the postictal period could be relevant in determining its length (and the number of apneic events).

These considerations must be integrated with a further finding, already widely documented for ICA,^11-13,20,21^ and recently also for postictal apnea due to amygdala stimulation,^20^ namely that patients are unaware of apneic phenomena. In this study, this phenomenon was evident not only for ictal apnea but also for postictal respiratory alterations. Indeed, none of our patients, even when explicitly questioned about it, ever expressed any awareness or distress about breathing alterations. Our findings must be interpreted considering that most seizures leading up to ICA/PICA originated from the temporal lobe, in which postictal confusion could also be a major confounding element in assessing patients' awareness of the apneic event. Moreover, peri-ictal amnesia could also partly explain the lack of recall of postictal apnea.

Postictal apnea has been well described by intracranial electrical stimulation of the amygdala^20^ with features very similar to those observed in focal seizures herein described. Noteworthy, previous studies of focal seizures in the Epilepsy Monitoring Unit setting have reported conflicting data. According to a previous study,^11^ postictal respiratory changes were present in only 3% of seizures with ICA and in 1% of all seizures recorded. These differences could be attributed to the diversity of the patients analyzed and, more probably, to the manner and “intensity” in testing the patient at the end of the seizure. One hypothesis could be that when the patient is tested very rapidly during and after the seizure resulting in a higher level of alertness and vigilance, the occurrence of postictal apnea is reduced or absent, unlikely when the patient is left alone, or during sleep (Figure 4). Testing this hypothesis would require a prospective study randomizing the intervention of the examiner in the postictal period, which we believe would be very difficult to perform.

Apnea occurring after a seizure (primary generalized or focal with tonic-clonic evolution ones) is now considered a risk factor of SUDEP.^5-8^ Regarding the potential risk of SUDEP in patients with ICA/PICA, we have no data that clearly associate peri-ictal respiratory changes in focal seizures with an increased risk of SUDEP. However, a case of SUDEP in a patient with ICA^19^ and a case of SUDEP in a patient with ICA/PICA induced by cerebral electrical stimulation^20^ have been previously reported. Therefore, the presence of ICA/PICA could represent a marker of fragility/susceptibility of brainstem respiratory centers in some patients, which need to be evaluated carefully.

A gap in knowledge is represented by the lack of studies that have investigated whether the presence of PICA in focal seizures is associated with postconvulsive apnea. To obtain these data, it would be necessary to investigate a large cohort of patients in whom both tonic-clonic seizures and focal seizures are recorded with cardiorespiratory polygraphy, to verify whether there is an association between the 2 phenomena (i.e., if patients with PICA are at risk of postconvulsive apnea). Unfortunately, in our population, we recorded only 5 focal seizures with tonic-clonic evolution and none in patients with ICA/PICA, which does not allow us to evaluate this hypothesis. A previous study^8^ investigated in detail the occurrence and features of postconvulsive apnea that was present in approximately 22% of all convulsive seizures (both in generalized convulsive seizures and in focal to bilateral tonic-clonic seizures). They observed that ICA occurrence did not predict postconvulsive apnea, but the occurrence of postictal apnea in focal seizures was not investigated.

Concerning our findings in postictal apnea after focal self-limiting seizures, we never observed an EEG postictal generalized suppression. This finding, in our opinion, underlines a relevant difference between persistent postictal apnea and postconvulsive apnea for which postictal EEG suppression has been frequently reported.^5-8^ Moreover, the fact that we never observed apneic events starting de novo in the postictal period in focal seizures suggests that ictal apnea persisting into the postictal period and postictal convulsive apnea likely reflect different underlying mechanisms.

Previous iEEG studies have consistently demonstrated that ictal apnea is related to the involvement of medial temporal lobe regions and of the amygdala by the ictal discharge.^13,14,16,25^ Furthermore, electrical stimulation of the amygdala has been shown to result in a time-locked respiratory arrest with the onset of stimulation lasting in some patients for several minutes after the end of stimulation.^13,15,17,20^ These studies testify how amygdala activity can bring about modifications (e.g., arrest) of the brainstem respiratory centers in humans.^20^ Although our study does not include patients with iEEG recordings, seizures with ICA and with PICA have been documented in patients with an anterior-mesial temporal epileptogenic zone in most of the cases. Furthermore, we documented an increased volume of the amygdala ipsilateral to the epileptogenic zone in patients with ICA/PICA compared with both healthy controls and patients with seizures without peri-ictal respiratory disturbances. Although this result does not have causal implications, it reinforces the possible role of this subcortical structure in the determinism of peri-ictal respiratory alterations. A hypertrophy of the amygdala has been reported previously by our group in patients with isolated ICA^31^ and recently in patients with focal to bilateral tonic-clonic seizures with postconvulsive apnea.^35^ Our work extends these findings by documenting how an amygdala enlargement is frequently present and may characterize patients with postictal apnea, even in focal nonconvulsive seizures. Thus, considering all the studies that have analyzed patients with peri-ictal apnea with volumetric imaging techniques, an enlarged amygdala has been documented in the following cohorts of patients: (1) ictal apnea in focal seizures, (2) postictal apnea in focal seizures, and (3) postconvulsive apnea.^35^ The totality of these data seems consistent with each other supporting the role of the amygdala in determining/fostering a variety of pericritical respiratory alterations. Although the pathologic changes related to amygdala volume increases have yet to be described, many different processes may underlie the documented changes, including gliosis, inflammation, and mTOR pathway activation causing neuronal architecture disruption.^36,37^ Indeed, recent data demonstrated increased mTOR pathway activity in postsurgery amygdala samples in patients with postconvulsive apnea, and ictal and postictal respiratory alterations have been documented in patients with focal epilepsy due to pathogenic *DEPDC5* variants.^21,38^

Apart from the fact that peri-ictal respiratory changes might be associated with an increased risk of SUDEP, a further element of alarm is the impact that repeated seizures with apnea and desaturation may have on patients' brain health, that is, repeated “small” hypoxic events may have regarding neuronal damage. Future studies should address this possibility, particularly through biomarkers of neurodegeneration or neuroaxonal damage after seizures with and without hypoxia. An interesting finding in this regard concerns previous studies documenting the presence of tissue biomarkers of hypoxia both in animals and in patients with drug-resistant temporal epilepsy.^39^

Certainly, this study has limitations. First, the observational nature of the study did not provide for standardization of the patient's testing/evaluation procedures in the postictal period, and the level of patient's awareness was not systematically addressed. Moreover, as already reported, we only recorded very few tonic-clonic seizures, which did not allow us to study possible associations between peri-ictal alterations in focal seizures and in seizures with tonic-clonic evolution. A possible limitation could be the lack of iEEG recordings, in the sense that one could speculate how the apnea episodes documented in the postictal period might be associated with persisting ictal activity at the amygdala level, which cannot be detected by scalp recordings. However, this hypothesis seems highly unlikely, and the fact that the apneic phenomena in the postictal period could be modified by external stimulation and vigilant interaction with an examiner seems to further render this possible limitation unlikely.

Our study, like previous ones, has not investigated directly EMG activity of respiratory muscles (intercostalis, diaphragm), and therefore, the evaluation of nature of ictal/postictal apneic events could be a further development in this area of research. Indeed, recent preclinical work in animal has documented how ictal/postictal apneic phenomena are associated with an absence of muscle activity in the diaphragm (atonic diaphragm).^40^

As far as neuroimaging analyses, the number of patients in the studied groups is certainly small. However, it is remarkable that despite the low numerosity of the samples, statistically significant differences have been found in the amygdala volumes. Certainly, the brain morphometry results need to be replicated in independent cohorts with larger sample sizes. Finally, it will be important to continue study significant differences in other subcortical structures that did not emerge in our study because of the small sample size.

Our data support the existence of a continuum from ictal to PICA and a key role of the amygdala, which shows increased volumes ipsilaterally to the epileptogenic zone. PICA occurs in approximately 6% of focal seizures and in 30% of focal seizures with ICA, even without secondary generalization and convulsion. Postictal apnea is associated with extended apnea time and prolonged oxygen desaturation representing a potential concern for chronic brain damage. Finally, this study highlights the importance of cardiorespiratory recordings in the Epilepsy Monitoring Units to assess seizure-related breathing disorders.

## Glossary

EMU: Epilepsy Monitoring Unit
FDR: false discovery rate
ICA: ictal central apnea
ICV: intracranial volume
iEEG: intracranial EEG
ILAE: International League Against Epilepsy
LTVEM: long-term video-EEG monitoring
PICA: postictal central apnea
SpO_2_: peripheral oxygen saturation
SUDEP: sudden unexpected death in epilepsy
TLE: temporal lobe epilepsy
